# Local Factors Determine Plant Community Structure on Closely Neighbored Islands

**DOI:** 10.1371/journal.pone.0019762

**Published:** 2011-05-10

**Authors:** Jianbo Lu, Lin Jiang, Lin Yu, Que Sun

**Affiliations:** 1 College of Life and Environmental Sciences, Xiasha Campus, Hangzhou Normal University, Hangzhou, Zhejiang Province, China; 2 School of Biology, Georgia Institute of Technology, Atlanta, Georgia, United States of America; 3 College of Life Sciences, Zijingang Campus, Zhejiang University, Hangzhou, Zhejiang Province, China; University of Zurich, Switzerland

## Abstract

Despite the recent popularity of the metacommunity concept, ecologists have not evaluated the applicability of different metacommunity frameworks to insular organisms. We surveyed 50 closely spaced islands in the Thousand-Island Lake of China to examine the role of local (environmental) and regional (dispersal) factors in structuring woody plant assemblages (tree and shrub species) on these islands. By partitioning the variation in plant community structure into local and regional causes, we showed that local environmental conditions, specifically island morphometric characteristics, accounted for the majority of the variation in plant community structure among the studied islands. Spatial variables, representing the potential importance of species dispersal, explained little variation. We conclude that one metacommunity framework–species sorting–best characterizes these plant communities. This result reinforces the idea that the traditional approach of emphasizing the local perspective when studying ecological communities continues to hold its value.

## Introduction

Understanding mechanisms regulating the structure of ecological communities is a central goal of community ecology. Until relatively recently, the prevailing opinion among ecologists is that species composition and abundance in a locality largely reflect species responses to local environmental conditions and the outcomes of species interactions, which are themselves influenced by local environmental conditions. This local perspective, which has its roots in the classic niche theory [1], [2], [3], [4], suggests that among-habitat differences in community structure are largely deterministic outcomes of differences in environmental conditions among habitats. A contrasting view emphasizes the importance of regional processes, particularly species dispersal, in determining community structure in localities [5], [6], [7], [8]. In its extreme case, this regional perspective is captured in neutral biodiversity models that assume functional equivalency among species across all habitats [8], [9], [10]. These neutral models suggest that among-habitat differences in community structure may simply arise from limited dispersal preventing species from reaching every habitat.

Most natural communities, however, are under the influence of both local (environmental) and regional (dispersal) factors [6], [7], [11], [12]. The metacommunity concept, defined as a system of local communities linked by dispersal [13], integrates local and regional processes in explaining community patterns. This concept suggests that the importance of regional processes relative to local processes varies with the frequency of species dispersal, resulting in different metacommunity frameworks [13]. As the metacommunity framework emphasizing regional processes, neutral models predict that dispersal limitation may result in metacommunities with distinct spatial structures, where neighboring habitats tend to share more similar species composition than distant habitats. As the metacommunity framework emphasizing local processes, the species sorting perspective predicts that the structure of communities approximates that determined by local conditions [13], [14]; this perspective goes beyond classic niche theory by explicitly acknowledging the necessity of sufficient levels of species dispersal for local regulation. On the other hand, the mass-effects perspective suggests that where dispersal is so frequent that it interferes with local community dynamics, community structure in a locality may deviate from that allowed by its environmental conditions [15], [16]. In particular, species that otherwise fail to persist in their unfavorable (sink) habitats may now be able to persist there as the result of considerable dispersal from their favorable (source) habitats. Finally, the patch-dynamics perspective emphasizes the importance of tradeoffs (e.g., competition-dispersal tradeoff) for species coexistence in the metacommunity, while also assuming uniform environmental conditions among local habitats as in the neutral perspective.

Empirical studies have revealed various types of metacommunities in nature [17]. For example, a number of researchers have reported that dispersal limitation influences tree species composition in tropical forests [18], [19], [20], others have shown that species sorting operated in a wide variety of taxa [21], [22], [23], [24], [25], [26]. A few studies also reported community patterns consistent with mass effects [22], [27], [28], [29] and patch dynamics [25], [30]. However, so far all empirical metacommunity studies have been implemented in aquatic systems such as ponds and lakes (e.g. [26], [27], [31]), or in terrestrial habitat patches and fragmented patches (e.g. [20], [21]). While these habitats may be considered as virtual islands, no studies, to our knowledge, have explored how different metacommunity frameworks apply to true insular communities.

Islands are important model systems for exploring various ecological questions [32], [33]. They are particularly suitable for answering metacommunity-related questions, because individual islands, embedded in aquatic landscapes unsuitable for most terrestrial organisms, have discrete boundaries that clearly define them as local habitats. Terrestrial organisms can actively (animals) or passively (plants) disperse among islands, resulting in the formation of potential island metacommunities. Insular biota, however, are generally less diverse than their mainland counterparts, facilitating the investigation of ecological mechanisms [33]. Here we tested the applicability of different metacommunity frameworks to plant communities on a cluster of closely neighbored islands in the Thousand-Island Lake of China. Given the close proximity of the study islands to one another and to the mainland (see below), which facilitates species dispersal, we hypothesized a predominant role of mass effects in structuring this island plant metacommunity. We identified the operating metacommunity framework by partitioning the variation [34] in plant community structure among the islands, with mass effects indicated by a significant spatial signature of the communities, independent of the variation in environmental conditions among the islands, as well as a significant effect of local environmental conditions on the communities, independent of the spatial structure of the communities [17].

## Materials and Methods

### Study area

The Thousand-Island Lake (hereafter TIL) is located in Chun'an county of Zhejiang Province, China (29°22′N to 29°50′N and 118°34′E to 119°15′E). It was created after the construction of Xin'an River Dam in 1958 for hydraulic electrical generation. TIL has a surface area of 583 km^2^, a volume of 17,840 km^3^, and an average depth of 34 m. A total of 1057 islands larger than 2500 m^2^ are present in the lake (hence its name), with the area of islands totaling 409 km^2^. The climate of the region is of sub-tropical monsoonal type, with average annual rainfall of 1429.9 mm and average annual temperature of 17°C. Vegetation was virtually absent on islands at the time of their formation due to extensive deforestation and conversion to agricultural lands in the 1950s; a 1964 survey showed that 46.7% of the TIL islands were still barren, 33.5% of the islands were colonized by early successional species (mainly Chinese red pine *Pinus massoniana*) and only 2.2% of the islands harbored later succesional evergreen broad-leaved forests [35]. This pattern suggests that seed banks probably played a limited role in the establishment of plants on the islands, and plants mainly colonized the islands from the nearby mainland (and presumably also from other islands acting as stepping stones) during the 50-year history of TIL [35], [36]. Current vegetation on these islands is dominated by Chinese red pine (*Pinus massoniana*), which, absent disturbance, will give way to evergreen broad-leaved forests dominated by Hardleaf Oatchestnut (*Castanopsis sclerophylla*) and Japanese blue oak (*Quercus glauca*) [37]. The major area of TIL has been designated as the National Forest Park (the largest in China), free from human disturbance, since 1986.

**Figure 1:**
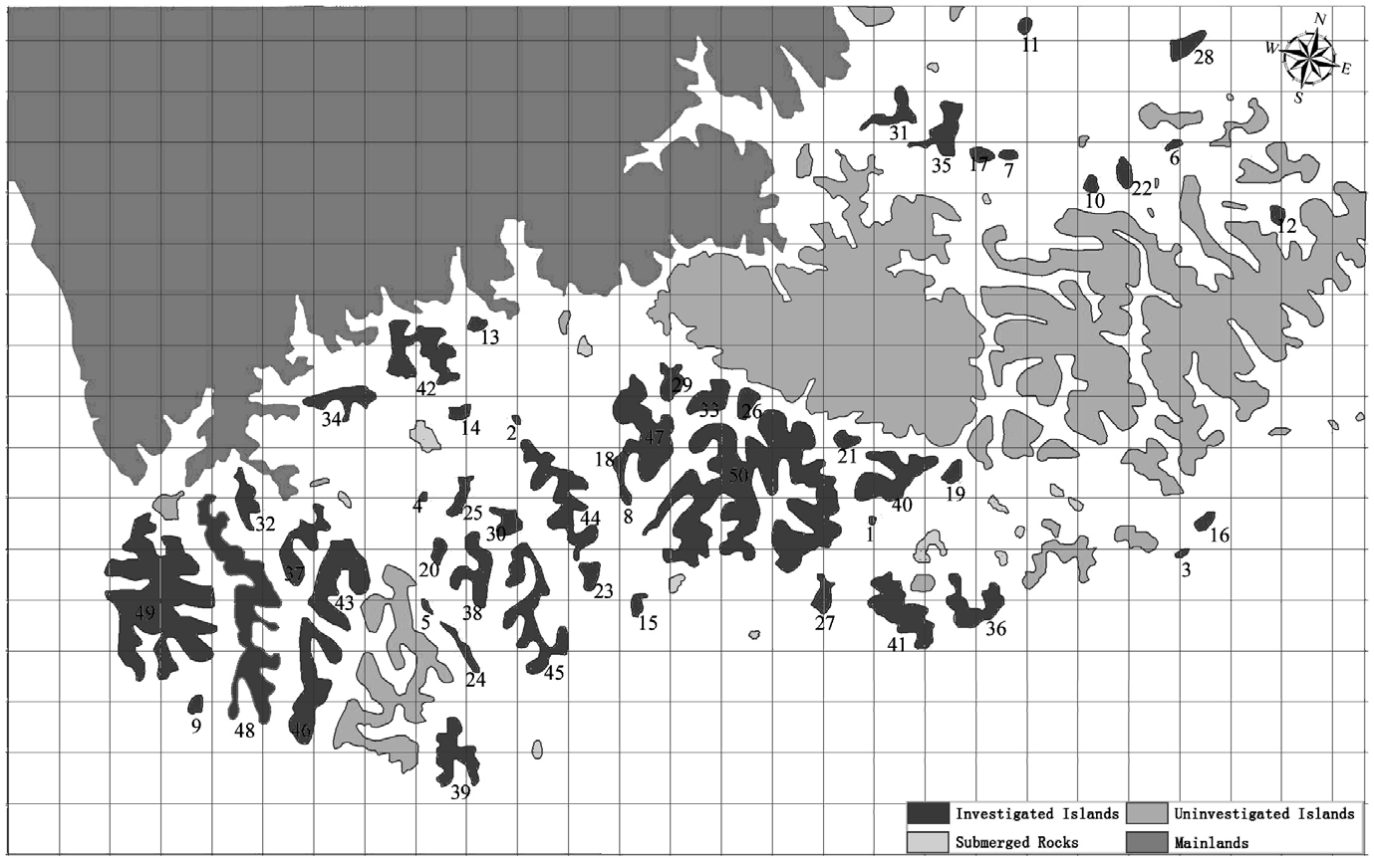
The spatial location of the 50 study islands in the TIL region. The grid in the map was used to help determine the location of the survey plots.

Our study area is located in the central part of the TIL region and includes 50 islands that are relatively close to each other, with the average distance to nearest neighboring islands 63.4 m (Fig. 1). The islands are also close to the mainland, with the average distance to the mainland 737.5 m. Among the 50 studied islands are 30 small islands (smaller than 1 ha), 17 medium islands (between 1 and 5 ha), and 3 large islands (larger 5 ha). The mean island area was 2.097 ha with a standard deviation of 3.75 ha.

### Plant survey

Our survey focused on trees and shrubs, the dominant plant forms on the TIL islands [38]. The survey took place from March to October in 2006. We used a grid-based sampling method where the study area was subdivided into grid cells of 200×200 m [39]. Within each grid, we established a plot of 30×30 m, with the plot location randomly selected on the island that falls within the grid [40]. For each of the 30 small islands, we surveyed tree species present in the designated plot, and then surveyed along the island ridge line as a supplementary method to record tree species not present in the plot. Using this approach, we were able to obtain the total count of every tree species on these small islands. We estimated the density of each shrub species, which was much more abundant than trees, in two 5×5 m sub-samples within the designated plot on the small islands. The same methods were used for islands of medium and large sizes, except that there were proportionally more sampling plots on these larger islands and that an exhaustive count of all individual trees was not possible.

### Data analysis

Our analysis focused on island morphometric variables, including island area, height, and shape as local environmental factors. We did not measure soil characteristics on the islands, where red soil is uniformly distributed [35], [41], [42], [43]. However, we acknowledge that soil heterogeneity (e.g., differences in soil nutrient and moisture) may exist within and among islands, and that the resultant habitat diversity may contribute to differences in plant community structure observed on these islands. We nevertheless expect island morphometric traits to capture at least some of the habitat diversity associated with soil heterogeneity (see Discussion). We used a digitized 1∶10000 topographical map to estimate the area, height, perimeter, distance to the mainland of each island, and distances among islands. Island shape index (S) was calculated as *S* = 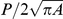 [44], where *P* is the island perimeter, and *A* is the island area; *S* is equal to 1 when the patch is circular and increases as patch becomes more irregular.

To discern the relative importance of local and regional factors for regulating plant community structure, we used variation partitioning [34] to divide among-island variation in plant composition and abundances into four fractions: a pure local (environmental) fraction explained by the spatially unstructured part of the environmental data, a pure regional (spatial) fraction independent of local environmental variation, a local-regional fraction explained by the spatially structured part of the environmental data, and unexplained variation. This was done by applying a redundancy analysis (RDA) to the plant abundance matrix that contained the abundance of each species on each island. We generated spatial predictors in the RDA using principal coordinate analysis of a truncated matrix of Euclidean distances among the islands [45], [46], [47], retaining the eigenvectors with only positive eigenvalues as the explanators. This approach is more effective than the commonly used polynomial trend surface analysis in capturing spatial patterns across a range of scales [45]. As the fractions of variation of the response matrix explained by the predictor matrices in canonical analyses are often biased, we adjusted each fraction in the RDA results following [48]. We conducted two RDAs with two types of spatial variables. In the first RDA, distances between islands were used to generate the spatial predictors to account for dispersal among islands. In the second RDA, distances between islands and the mainland were used to account for dispersal from the mainland to the islands. The statistical significance of fractions was tested according to [48], using 4,999 permutations.

## Results

There were 54 tree species and 67 shrub species found on the surveyed islands, totaling 121 woody species. The number of tree and shrub species on the islands ranged from 3 to 24 and from 3 to 34, respectively; the total number of woody species ranged from 9 to 55. Larger islands tend to contain more species than smaller islands (Table S1). Many tree species (e.g., *Pinus massoniana*, *Quercus fabri*, *Symplocos paniculata*) and shrub species (e.g., *Rhododendron simsii*, *Loropetalum chinense*, *Smilax davidiana*, *Grewia biloba*) were widely distributed across islands of different sizes. However, a number of other trees (e.g., *Mallotus apelta*, *Ilex chinensis*, *Broussonetia papyrifera*) and shrubs (e.g., *Syzygium buxifolium*, *Smilax china*, *Ilex cornuta*) can only be found on intermediate and large islands. The only exception to this nested distribution pattern is the shrub *Symplocos sumuntia*, which was present only on smaller islands.

**Figure 2:**
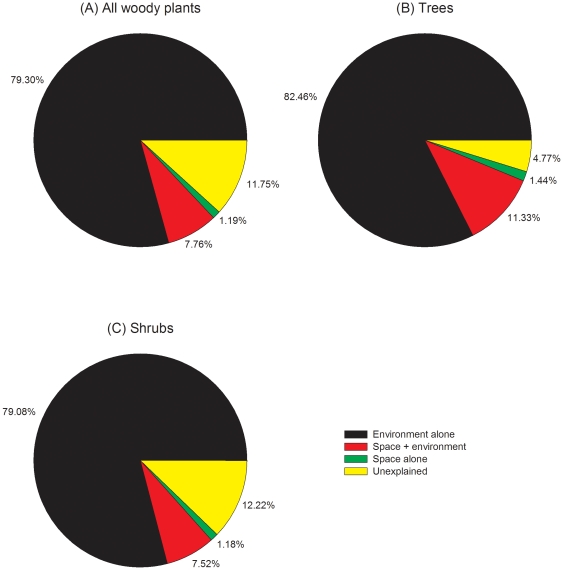
Results of variation partitioning of woody plant community structure on the 50 TIL islands using RDA, in which spatial predictors were generated using distance-based eigenvector maps based on among-island distances. (A) All woody species, (B) tree species, and (C) shrub species.

Variation partitioning based on distances between islands revealed that only pure environmental variables were significant in explaining variation in plant community structure on the 50 TIL islands (Fig. 2). Pure local environmental variables accounted for approximately 80% (P<0.001 in all three cases) of the total variation in community structure for all woody species considered together (trees and shrubs combined, Fig. 2A), and for trees (Fig. 2B) and shrubs (Fig. 2C) considered separately. By contrast, the non-significant pure spatial predictors never explained more than 1.5% of the total variation, suggesting the little role of among-island species dispersal in regulating plant community structure on these islands. As a result, pure local environmental variables explained a significantly greater portion of total variance than pure spatial variables (P<0.0001).

**Figure 3:**
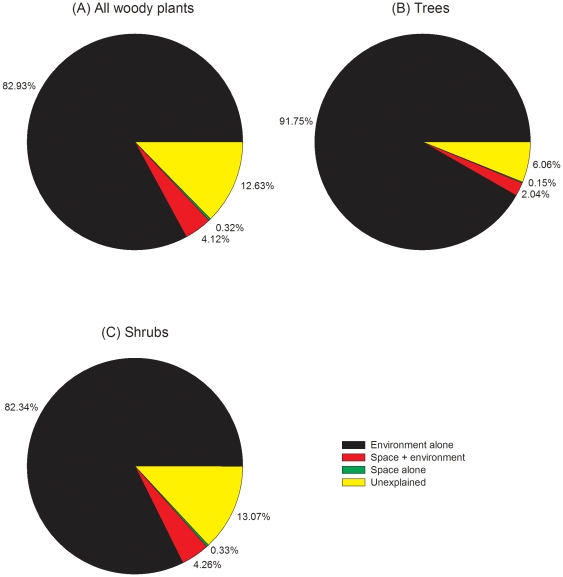
Results of variation partitioning of woody plant community structure on the 50 TIL islands using RDA, with spatial predictors based on distances to the mainland. (A) All woody species, (B) tree species, and (C) shrub species.

Strikingly similar results were found when variance partitioning was based on island distances to the mainland (Fig. 3). Again pure local environmental variables were the only significant predictor, explaining more than 80% (P<0.001 in all three cases) of the total variation in community structure of all woody plants considered together (Fig. 3A), and for trees (Fig. 3B) and shrubs (Fig. 3C) considered separately. Here the non-significant pure spatial predictors explained no more than 0.33% of the total variation, indicating the little role of species dispersal from the mainland in determining plant community structure on these islands. Pure local environmental variables again explained a significantly greater portion of total variance than pure spatial variables (P<0.0001).

## Discussion

The close proximity of our study islands to one another and to the mainland led us to make the a priori prediction that mass effects, whereby high levels of species dispersal alter the impacts of local environmental conditions on community structure, is likely to characterize this insular plant metacommunity. Our results, however, do not support this prediction. The majority of variation in plant community structure can be attributed to variation in local environmental conditions, with little evidence for the role of pure spatial effects. In fact, the percentages of variation in community structure explained by the pure environmental (ca. 80%) and spatial (less than 1.5%) components are, respectively, among the largest and smallest that have been reported [17], suggesting a tight local regulation of woody plant communities on these islands. This scenario can be best depicted by the species sorting framework that recognizes the predominant structuring role of local environmental effects [13], [14].

Species sorting has been found to be important in a variety of systems, including temperate forest trees [21], invertebrates and amphibians in ponds [22], zooplankton in interconnected ponds [23], invertebrates in rock pools [24], mosquitoes in water-filled tree holes [25], and aquatic bacterial communities spanning a wide range of spatial scales [26]. In particular, Cottenie and De Meester [23] showed that in a system of highly interconnected ponds, patterns in zooplankton composition and diversity were largely determined by individual pond environmental characteristics and thus conformed to the species sorting perspective. This is despite frequent zooplankton dispersal among the ponds [49], which presumably promotes mass effects. This result prompted Cottenie and De Meester [23] to conclude that zooplankton communities in many lakes and ponds should exhibit patterns consistent with species sorting. Likewise, our results suggest that patterns of plant communities on many islands, including closely spaced islands such as those examined here, may also conform to the species sorting perspective. This, of course, does not exclude the possibility that dispersal limitation may shape plant communities on islands that are further away from the mainland and much more distantly spaced from one another. It is thus likely that the importance of dispersal limitation relative to species sorting may increase with increasing spatial scales, an idea that can be tested by expanding the scale of our analysis to include more distant islands in the TIL region.

Note that for species sorting to occur, dispersal must be sufficiently frequent so that species suitable for certain habitats are able to colonize these habitats. Common mechanisms of plant seed dispersal, with wind, water, and animals as dispersal agents [50], all likely operate in the TIL region. Seeds with wing- and plume-like structures, such as those produced by the Chinese guger tree (*Schima superba*), Chinese red pine (*Pinus massoniana*), and Beautiful Sweetgum (*Liquidambar formosana*), can readily move via aerial and water transport among the TIL islands. Animal-aided dispersal, with insects, birds, and mammals as vectors, may also play a significant role in the plant colonization of the TIL islands. In particular, dispersal by birds, which are abundant in the TIL region, may be important for transporting seeds of a large number of plant species across islands. For example, the seeds of Chinese Pistachios (*Pistacia chinensis*), a common tree on many islands in the TIL region, can be transported at an average distance of 300–500 m by birds [51], [52]. Mammals present in the TIL region, such as wild rabbits (*Lepus sinensis formosus*), Chinese muntjac (*Muntiacus reevesi*), and wild pigs (*Sus scrofa*), can also transport seeds in the same way when they swim between islands to find food and mates.

Three island morphometric variables, including island size, height, and shape, accounted for the majority of variation in the structure of plant communities among the study islands. Additional RDAs, with only one of the three variables entered as the environmental factors, produced qualitatively similar results. These additional analyses also showed that while each island variable explained considerable, statistically significant, variation, island size was the single better explanator than island height and shape. We suggest that the ability of these variables in determining plant community structure likely reflects their influences on habitat diversity on the islands, with island size possibly exerting the largest influence. Increasing island size and height tend to increase spatial heterogeneity in the availability of sunlight, water, and soil, translating into more different kinds of microhabitats suitable for different species. For example, whereas cold- and shade-resistant species (e.g. Gardenia [*Gardenia jasminoides*]) can grow on the north-facing slope of large, tall islands, the sunny south-facing slope on these islands often harbors a different set of plant species (e.g., Beautiful Sweetgum [*Liquidambar formosana*], Crow Persimmon [*Diospyros kaki* var. *silvestris*]) that are less resistant to cold or shade stress. Also thick soil layers with high moisture content are typically found in the valleys, contrasting with the typical thin soil layers with low moisture content along island ridge lines. This difference in microhabitats again favors different species: the Chinese Pistachios (*Pistacia chinensis*), for example, is generally restricted to the valley microhabitats. Island height also affects the magnitude and frequency of disturbance events associated with changes in the TIL water level: islands with smaller statures are more likely to be submerged when water level rises, and are hence increasingly dominated by flood-tolerant plants (e.g. Chinese red pine [*Pinus massoniana*], Chinese tallow tree [*Sapium sebiferum*]). Lastly, island shape may also affect the distribution of microhabitats of the islands. In particular, irregularly shaped islands tend to contain large edge habitats but small interior habitats, and the relative abundance of plant species preferring these two different habitats would change with island geometry. Previous research has shown that habitat geometry affects plant species diversity [53] and relative abundance [54].

We have shown here that island morphometric characteristics largely determine the structure of woody plant communities on a cluster of closely spaced lake islands. An important caveat of our work is that we did not measure soil physical and chemical properties known to affect plant growth, and hence cannot determine the contribution of habitat diversity associated with soil heterogeneity to variation in plant community structure among the islands. Although the effect of soil heterogeneity was, to a certain extent, represented in the island morphometric variables, explicitly including both island soil and morphometric properties in the analysis would help disentangle their roles in regulating plant communities on these islands. Despite this caveat, our result demonstrates the importance of local environmental regulation in a true insular system, adding to the growing evidence that species sorting may be the dominant metacommunity framework that characterizes many natural systems [14], [17]. More investigations on the applicability of different metacommunity frameworks in understudied ecosystems, such as the island ecosystems examined here, are needed. More importantly, we suggest that an essential next step in metacommunity research is to not only characterize metacommunity patterns but also elucidate mechanisms underlying the observed patterns (e.g., those determining the relative importance of local and regional factors in regulating communities; see [55], [56], [57]). For the TIL islands, the question is then why species sorting dominates despite considerable dispersal? Experimental manipulation of both species dispersal and environmental conditions may help answer this question (e.g. [58]).

## Supporting Information

Table S1 The area, height, and plant species richness of the surveyed islands. Islands are ranked by area (the smallest island ranked the first).
(DOC)
