# Supplementary material for: Local Factors Determine Plant Community Structure on Closely Neighbored Islands
Source: PLoS One. 2011 May 10;6(5):e19762. doi: 10.1371/journal.pone.0019762 (PMC3091879; doi:10.1371/journal.pone.0019762)
Supplement: Table S1 — The area, height, and plant species richness of the surveyed islands. Islands are ranked by area (the smallest island ranked the first). (DOC) [file pone.0019762.s001.doc]

**Supporting Information**

Table S1. The area, height, and plant species richness of the surveyed islands. Islands are ranked by area (the smallest island ranked the first).

| Island number | Area (ha) | Height (m) | Species richness of trees | Species richness of shrubs | Woody species richness |
| --- | --- | --- | --- | --- | --- |
| 1 | 0.077 | 2.0 | 5 | 9 | 14 |
| 2 | 0.090 | 2.0 | 6 | 3 | 9 |
| 3 | 0.093 | 2.9 | 4 | 9 | 13 |
| 4 | 0.118 | 5.0 | 8 | 12 | 20 |
| 5 | 0.136 | 6.0 | 4 | 10 | 14 |
| 6 | 0.186 | 7.0 | 3 | 7 | 10 |
| 7 | 0.237 | 2.0 | 9 | 17 | 26 |
| 8 | 0.264 | 7.0 | 15 | 17 | 32 |
| 9 | 0.268 | 7.0 | 14 | 18 | 32 |
| 10 | 0.289 | 8.0 | 9 | 18 | 27 |
| 11 | 0.306 | 4.0 | 12 | 18 | 30 |
| 12 | 0.312 | 5.0 | 10 | 19 | 29 |
| 13 | 0.325 | 3.3 | 4 | 9 | 13 |
| 14 | 0.332 | 4.0 | 9 | 14 | 23 |
| 15 | 0.393 | 6.0 | 9 | 13 | 22 |
| 16 | 0.418 | 8.0 | 6 | 15 | 21 |
| 17 | 0.442 | 9.0 | 14 | 22 | 36 |
| 18 | 0.443 | 11.0 | 10 | 16 | 26 |
| 19 | 0.456 | 8.0 | 7 | 15 | 22 |
| 20 | 0.466 | 7.0 | 10 | 16 | 26 |
| 21 | 0.485 | 10.0 | 17 | 24 | 41 |
| 22 | 0.563 | 15.0 | 5 | 21 | 26 |
| 23 | 0.590 | 14.8 | 13 | 17 | 30 |
| 24 | 0.617 | 6.0 | 7 | 13 | 20 |
| 25 | 0.635 | 5.0 | 9 | 11 | 20 |
| 26 | 0.689 | 21.0 | 11 | 21 | 32 |
| 27 | 0.736 | 11.0 | 11 | 19 | 30 |
| 28 | 0.842 | 17.0 | 13 | 22 | 35 |
| 29 | 0.889 | 10.0 | 13 | 27 | 40 |
| 30 | 0.957 | 12.0 | 11 | 14 | 25 |
| 31 | 1.091 | 14.0 | 11 | 15 | 26 |
| 32 | 1.183 | 22.0 | 13 | 19 | 32 |
| 33 | 1.372 | 13.0 | 15 | 24 | 39 |
| 34 | 1.598 | 15.8 | 18 | 23 | 41 |
| 35 | 1.750 | 17.0 | 15 | 21 | 36 |
| 36 | 2.204 | 29.0 | 15 | 26 | 41 |
| 37 | 2.204 | 22.0 | 17 | 24 | 41 |
| 38 | 2.232 | 18.2 | 11 | 14 | 25 |
| 39 | 2.354 | 12.0 | 16 | 28 | 44 |
| 40 | 3.345 | 22.0 | 16 | 27 | 43 |
| 41 | 3.399 | 36.0 | 14 | 26 | 40 |
| 42 | 3.541 | 16.8 | 18 | 23 | 41 |
| 43 | 4.089 | 28.5 | 11 | 23 | 34 |
| 44 | 4.155 | 21.0 | 21 | 30 | 51 |
| 45 | 4.173 | 21.0 | 18 | 32 | 50 |
| 46 | 4.254 | 36.8 | 14 | 24 | 38 |
| 47 | 4.835 | 42.0 | 22 | 25 | 47 |
| 48 | 8.744 | 35.7 | 20 | 27 | 47 |
| 49 | 13.889 | 67.0 | 24 | 31 | 55 |
| 50 | 21.785 | 49.0 | 20 | 34 | 54 |
| Mean | 2.097 | 15.5 | 12.14 | 19.24 | 31.38 |
